# Comparison of multivariable logistic regression and machine learning models for predicting pharyngocutaneous fistula after surgery for laryngeal and hypopharyngeal carcinoma

**DOI:** 10.3389/fsurg.2026.1867850

**Published:** 2026-09-03

**Authors:** Xiaoqin Ji, Huiling Zhao

**Affiliations:** 1 Department of Otolaryngology Head and Neck Surgery, West China Hospital, Sichuan University, Chengdu, China; 2 West China School of Nursing, Sichuan University, Sichuan Province, Chengdu, China

**Keywords:** hypopharyngeal carcinoma, laryngectomy, logistic regression, machine learning, pharyngocutaneous fistula

## Abstract

**Purpose:**

The aim of this study was to comprehensively compare multifactorial logistic regression and various machine learning models in predicting pharyngocutaneous fistula (PCF) following laryngectomy in patients with laryngeal carcinoma and hypopharyngeal carcinoma.

**Methods:**

Utilizing a significant dataset from West China Hospital, Sichuan University, we retrospectively analyzed the medical records of 2,863 patients diagnosed with laryngeal or hypopharyngeal cancer who underwent surgical treatment from 17 March 2008 to 9 May 2022 to identify critical risk factors for postoperative PCF. Our approach encompassed traditional statistical methods and advanced machine learning techniques, including Random Forest, Decision Tree, XGBoost, and Support Vector Classification.

**Results:**

Of the 2,863 patients undergoing laryngectomy, 263 (9.18%) developed postoperative PCF. In the validation set, the XGBoost model achieved the highest AUC (0.759), while the multivariable logistic regression model achieved an AUC of 0.753; however, the difference was not statistically significant. Logistic regression showed favorable calibration and clinical net benefit and was selected as the final model. Skin flap reconstruction and advanced tumor stage, particularly T3/T4 and N2/N3 disease, were important predictors of PCF.

**Conclusion:**

Machine learning models showed predictive performance comparable to multivariable logistic regression but did not significantly improve discrimination. Considering its interpretability, calibration, clinical net benefit, and ease of implementation, multivariable logistic regression may be a practical model for predicting postoperative PCF after laryngectomy. Further prospective studies with external validation are warranted.

## Introduction

Pharyngocutaneous fistula (PCF) is a common postoperative complication after laryngectomy for laryngeal or hypopharyngeal carcinoma. It is characterized by an abnormal communication between the pharyngeal or cervical esophageal lumen and the skin, resulting in leakage of saliva or other luminal secretions through the surgical wound (1–3). Recent literature has reported an incidence rate of 3%–65% (4–6). The ensuing persistent neck wound infection can provoke life-threatening vascular ruptures, causing major hemorrhages (7) and jeopardizing patient safety. Moreover, the development of PCFs can delay wound healing, impede the recovery of vocalization and swallowing functions, and postpone the optimal timing for adjuvant radiotherapy and chemotherapy, significantly affecting disease outcomes.

PCF is a potentially preventable complication, and early identification of patients at high risk may facilitate targeted preventive interventions. In recent years, machine learning (ML) approaches have increasingly been applied to disease -risk prediction and the identification of important predictive features in large clinical datasets, whereas logistic regression (LR) remains one of the most widely used multivariable models in medical research (8). Previous studies have used multivariable LR analyses to identify factors associated with PCF after laryngectomy (9, 10), and ML methods have also been used to identify important predictors of PCF (7). However, few studies have directly compared the predictive performance of multivariable LR and ML models for PCF occurrence. Moreover, large-scale studies developing and validating PCF risk prediction models in Chinese populations remain limited.

Therefore, this study aimed to develop and compare multivariable logistic regression and machine learning models for predicting the occurrence of PCF after laryngectomy in patients with laryngeal or hypopharyngeal carcinoma. We evaluated and compared their discrimination and calibration to determine whether machine learning approaches provided additional predictive value over multivariable logistic regression for PCF risk prediction.

## Materials and methods

### Study subjects

**Figure 1:**
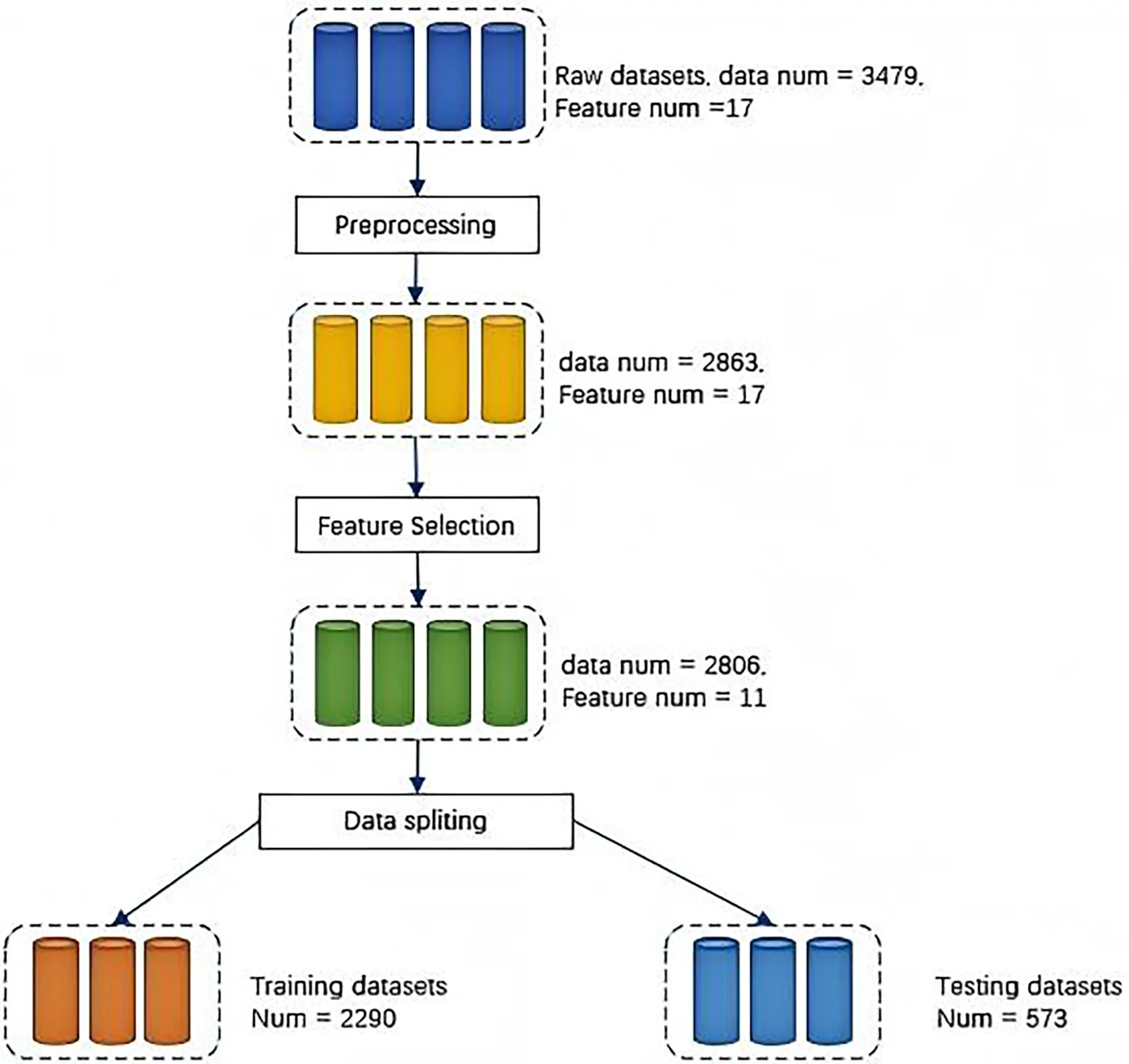
Patient selection flow diagram.

This retrospective study reviewed medical records of patients diagnosed with laryngeal or hypopharyngeal cancer who underwent surgical treatment at the Department of Otolaryngology–Head and Neck Surgery, West China Hospital of Sichuan University, between 17 March 2008 and 9 May 2022. Ethical approval was granted by the Institutional Review Board (IRB) of West China Hospital, Sichuan University, under the reference number 20221760. During the study period, a total of 3,479 patients were initially included. After the exclusion of missing, invalid, or inconsistent tumor, node, and metastasis (TNM) staging records, as detailed in Figure 1, the final analysis comprised data from 2,863 patients, with a PCF incidence of 9.18%. PCF was defined as clinically evident leakage of saliva or pharyngeal secretions through the cervical wound due to an abnormal communication between the pharyngeal or cervical esophageal lumen and the skin.

### Prediction time point and predictor eligibility

This study aimed to develop a model for early postoperative risk stratification at the end of surgery. Candidate predictors were therefore restricted to variables available before or at the completion of surgery, including preoperative and intraoperative variables. Variables occurring after surgery or those potentially influenced by PCF, such as length of hospitalization, were excluded to prevent information leakage and avoid overestimation of model performance. The eligible predictors were selected exclusively within the training set using the procedure described in the following, whereas the validation set was reserved for final model evaluation.

### Variable selection

Risk factors for PCF, as reported in previous literature, were collected and combined with clinical expertise to design a survey on factors influencing the risk of PCF occurrence after laryngectomy. Data were retrieved from the hospital's electronic medical record system and included the following aspects: (i) basic patient information at the time of admission included age, gender, body mass index (BMI), smoking, and drinking habits; (ii) disease characteristics: tumor staging [based on the 2017 International Union Against Cancer (UICC) standards], the number of surgeries, comorbidities such as hypertension and diabetes, radiotherapy and chemotherapy status, and bleeding events; and (iii) surgical factors: concomitant neck dissection, skin flap reconstruction, preoperative use of antibiotics, and duration of surgery. For clarity, neck dissection refers to patients with laryngeal or hypopharyngeal cancer who underwent open neck surgery (including salvage laryngectomy). Preoperative antibiotics refer to the intravenous administration of antibiotics 30 min before the start of the surgery.

### Statistical analysis

Continuous variables were analyzed using the Mann–Whitney U test, while categorical variables were compared using Fisher's exact test. To identify factors independently associated with PCF, we performed univariable and multivariable logistic regression analyses. First, we performed univariate analysis and selected the clinical variables with a *P*-value of less than 0.05. Then, we performed multivariate analysis with those factors. Analyses were conducted using SPSS for Windows ver. 20.0 (SPSS Inc., Chicago, IL, USA). Results were presented as odds ratios (ORs) with 95% confidence intervals (CIs), and a *P*-value of less than 0.05 was considered statistically significant.

### Logistic regression analysis

Univariable logistic regression was initially performed to evaluate associations between the candidate variables and the development of PCF after laryngectomy. The following variables were significantly associated with PCF in the univariable analyses (*P* < 0.05): neck dissection, skin flap reconstruction, number of operations, radiotherapy, chemotherapy, diabetes, intraoperative blood loss, T stage, N stage, smoking, preoperative antibiotic use, and duration of operation. These variables were subsequently entered into the multivariable logistic regression model.

In the multivariable logistic regression analysis, with postoperative PCF as the dependent variable, neck dissection, skin flap reconstruction, number of operations, diabetes, T3 stage, T4 stage, N2 stage, N3 stage, and duration of operation were independently associated with an increased risk of PCF after laryngectomy. The detailed results of the univariable and multivariable analyses are presented in Table 1.

**Table 1:** Multivariable logistic regression analysis of the risk of PCF in laryngeal cancer patients after surgery.

Variables	Beta	SE	Z	OR (95% CI)	*P*	aBeta	aSE	aZ	aOR (95%CI)	adjust-*P*
Neck dissection										
No				1.00 (Reference)					1.00 (Reference)	
Yes	−0.90	0.14	−6.59	0.41 (0.31–0.53)	<0.001	0.58	0.22	2.66	1.78 (1.16–2.72)	0.008
Skin flap reconstruction										
No				1.00 (Reference)					1.00 (Reference)	
Yes	1.87	0.15	12.90	6.52 (4.90–8.67)	<0.001	1.31	0.17	7.60	3.70 (2.64–5.18)	<0.001
Number of operations										
1				1.00 (Reference)					1.00 (Reference)	
≥1	0.88	0.16	5.64	2.41 (1.78–3.28)	<0.001	0.76	0.17	4.39	2.14 (1.52–3.02)	<0.001
Chemoradiotherapy										
No				1.00 (Reference)					1.00 (Reference)	
Yes	1.05	0.18	5.71	2.87 (2.00–4.11)	<0.001	0.44	0.21	2.13	1.55 (1.04–2.33)	0.033
Age										
<60 years				1.00 (Reference)						
≥60 years	−0.20	0.13	−1.49	0.82 (0.63–1.06)	0.137					
Diabetes										
No				1.00 (Reference)					1.00 (Reference)	
Yes	0.57	0.21	2.74	1.76 (1.18–2.64)	0.006	0.68	0.23	3.00	1.98 (1.27–3.08)	0.003
Hypertension										
No				1.00 (Reference)						
Yes	0.21	0.17	1.27	1.24 (0.89–1.72)	0.205					
BMI										
<18.5				1.00 (Reference)						
18.5–24.9	−0.22	0.19	−1.11	0.81 (0.55–1.18)	0.265					
≥24.9	−0.42	0.23	−1.86	0.66 (0.42–1.02)	0.063					
Bleeding										
<100 mL				1.00 (Reference)					1.00 (Reference)	
≥100 mL	0.97	0.15	6.29	2.64 (1.95–3.58)	<0.001	0.09	0.19	0.50	1.10 (0.76–1.60)	0.619
Smoking										
No				1.00 (Reference)					1.00 (Reference)	
Yes	0.56	0.17	3.31	1.74 (1.25–2.42)	<0.001	0.56	0.19	2.98	1.76 (1.21–2.54)	0.003
Drinking										
No				1.00 (Reference)						
Yes	−0.02	0.14	−0.12	0.98 (0.74–1.30)	0.904					
Preoperative antibiotics										
No				1.00 (Reference)					1.00 (Reference)	
Yes	−1.20	0.19	−6.26	0.30 (0.21–0.44)	<0.001	−0.60	0.27	−2.26	0.55 (0.32–0.92)	0.024
Duration of surgery										
<1 h				1.00 (Reference)					1.00 (Reference)	
1–2 h	0.44	0.19	2.33	1.55 (1.07–2.24)	0.020	−0.34	0.26	−1.35	0.71 (0.43–1.17)	0.178
≥2 h	1.25	0.19	6.76	3.50 (2.43–5.04)	<0.001	−0.38	0.29	−1.31	0.68 (0.39–1.21)	0.190
T stage										
T1				1.00 (Reference)					1.00 (Reference)	
T2	0.65	0.26	2.56	1.92 (1.16–3.17)	0.011	0.49	0.27	1.85	1.64 (0.97–2.77)	0.065
T3	1.66	0.25	6.57	5.27 (3.21–8.65)	<.001	1.18	0.29	4.14	3.26 (1.86–5.70)	<0.001
T4	1.92	0.24	7.93	6.82 (4.24–10.95)	<.001	1.15	0.28	4.05	3.16 (1.81–5.51)	<0.001
N stage										
N0				1.00 (Reference)					1.00 (Reference)	
N1	0.89	0.18	5.03	2.43 (1.72–3.42)	<0.001	0.48	0.24	1.96	1.62 (1.00–2.61)	0.050
N2	1.37	0.16	8.39	3.94 (2.86–5.42)	<0.001	0.84	0.25	3.41	2.31 (1.43–3.74)	<0.001
N3	2.13	0.28	7.70	8.39 (4.88–14.40)	<0.001	1.24	0.36	3.46	3.44 (1.71–6.93)	<0.001
M stage										
M0				1.00 (Reference)						
M1	1.01	0.57	1.77	2.75 (0.90–8.43)	0.076					

β, unadjusted regression coefficient from the univariable analysis; aβ, adjusted regression coefficient from the multivariable logistic regression model; SE, standard error; OR, odds ratio; CI, confidence interval. Positive coefficients (β or aβ) indicate higher odds of PCF occurrence, whereas negative coefficients indicate lower odds, compared with the reference category. OR = exp(β) or exp(aβ); for categorical variables, comparisons are made with the specified reference category. Unadjusted *P-*values (*P*) and adjusted *P*-values (adjust-*P*) are presented; values <0.05 were considered statistically significant.

### Data partitioning and sample size determination

The entire dataset consisted of 2,863 patients and was randomly stratified into a training set and a validation set based on the distribution of the outcome variable (complication). The training set comprised 2,290 patients (80% of the entire dataset), whereas the validation set comprised 573 patients (20%). The training set was used for data preprocessing, feature selection, model development, and hyperparameter optimization, while the validation set was used to evaluate model performance during model development.

### Data preprocessing and feature selection

For categorical variables with no ordinal relationship (e.g., smoking, drinking, T stage, N stage), one-hot encoding was employed to transform each category into a distinct binary feature. This prevented the AI algorithms from misinterpreting nominal categories as ordinal integer values.

To handle missing data in variables such as “Smoking,” “Drinking,” “Preoperative antibiotics,” and “Duration of surgery,” we employed a random forest (RF)-based imputation approach. In particular, a predictive random forest model was constructed using preoperative and intraoperative predictors available at the time of surgery (e.g., age, BMI, neck dissection, lymph node dissection, skin flap reconstruction, operation number, preoperative radiotherapy, diabetes, hypertension, intraoperative blood loss, T, N, and M stage) as input features. Crucially, postoperative variables such as days of hospitalization were excluded from the imputation model to avoid information leakage. The random forest imputation utilized the variable importance ranking to predict missing values. The robustness of each imputation model was validated via 10-fold cross-validation within the training set, and predictive accuracy was assessed using appropriate metrics, such as the root mean square error (RMSE) or mean absolute error (MAE).

### Machine learning model development

After feature engineering and imputation, multicollinearity among all candidate predictor variables was assessed using the variance inflation factor (VIF), with a threshold of VIF ≤10 applied for variable retention. All 17 identified candidate predictors met this criterion and were input into five modeling algorithms: LR, RF, Decision Tree (DT), XGBoost (XGB), and support vector machine (SVM). Five machine learning models were constructed, with tailored optimization strategies implemented to mitigate overfitting.

#### Logistic regression

A binary logistic regression model was constructed using all features as independent variables and complication as the dependent variable, with parameters estimated via maximum likelihood estimation.

#### Random forest

An ensemble classification model was built using bootstrap sampling. To control overfitting risk, the following optimization strategies were applied: (1) automatic selection of the optimal number of randomly selected features per node (mtry) using the tuneRF function; (2) the number of trees set to 1,000; (3) minimum node size increased to 20; (4) maximum number of nodes per tree limited to 30; and (5) 70% of training samples used for bootstrap resampling. Variable importance was evaluated using the Gini impurity reduction metric.

#### XGBoost

A gradient-boosting decision tree ensemble model was constructed with multiple regularization strategies to enhance generalizability: (1) learning rate set to 0.03; (2) maximum tree depth limited to 4; (3) minimum child weight set to 5; (4) subsample ratio set to 80%; (5) column subsampling ratio set to 70%; (6) simultaneous incorporation of L1 regularization (*α* = 1) and L2 regularization (*λ* = 2); and (7) minimum loss reduction threshold (*γ* = 0.1). Ten-fold cross-validation with early stopping (early stopping rounds = 50) was employed to automatically determine the optimal number of boosting rounds.

#### Decision tree

A classification tree was constructed using recursive partitioning, with cost complexity pruning applied to prevent overfitting. Initial tree parameters were set as follows: minimum node size of 20, minimum leaf node size of 7, complexity parameter (CP) of 0.01, and maximum depth of 10. The optimal CP was selected via 10-fold cross-validation to minimize cross-validated error, and the initial tree was pruned accordingly.

#### Support vector machine

A non-linear classifier was constructed using the radial basis function kernel. Hyperparameters—including the penalty parameter C (search range: 10⁻^1^ to 10^2^) and kernel parameter *γ* (search range: 10⁻^3^ to 10^1^)—were optimized via grid search with 10-fold cross-validation. Model training was performed with a convergence tolerance of 0.001, and the shrinking heuristic strategy was enabled to improve computational efficiency.

To ensure methodological rigor and prevent information leakage, all feature selection procedures (VIF-based multicollinearity assessment) and hyperparameter optimization (including automated mtry selection, grid search, cross-validation-based early stopping, and cost complexity pruning) were performed exclusively within the training set. The independent validation set was not exposed to any model development or tuning steps, ensuring unbiased performance evaluation. Detailed hyperparameter configurations for each algorithm are provided in Supplementary Table S1.

### Model evaluation

Model performance was comprehensively evaluated using the area under the receiver operating characteristic curve (AUC-ROC), sensitivity, specificity, accuracy, Brier score, and calibration plots. The clinical utility of each model was further assessed using decision curve analysis (DCA). DeLong tests were performed to compare the AUC differences between models. Based on the trade-off between predictive accuracy, calibration stability, generalizability, and clinical net benefit, we ultimately prioritized the logistic regression model despite the theoretical advantages of machine learning in capturing non-linear interactions.

### Reporting guidelines and software

All statistical analyses were conducted using statistical software R (version 4.0.5) and Python (version 3.7) with relevant libraries (e.g., pandas, scikit-learn, xgboost). The reporting of this study adheres to the TRIPOD + AI (Transparent Reporting of a multivariable prediction model for Individual Prognosis or Diagnosis—Artificial Intelligence) guidelines, ensuring methodological transparency and reproducibility.

## Results

### Clinical and Pathological Characteristics of Patients

The baseline data for patients with PCF and those without are presented in Table 2. Among the 2,863 patients included, 263 (9.18%) developed PCF. There were no statistically significant differences in patient characteristics and preoperative variables in the training and testing sets (Table 2).

**Table 2:** Baseline data of laryngeal cancer patients with and without PCF after surgery.

Characteristics	PCF yes (n1 = 263)	PCF no (n2 = 2,600)	Total (*N* = 2,863)	*P*-value
Neck dissection				<0.001
Yes	234 (8.17%)	1,783 (62.28%)	2,017 (70.45%)	
No	0 (0%)	846 (29.55%)	846 (29.55%)	
Lymph node dissection				<0.001
No	94 (3.28%)	1,505 (52.57%)	1,599 (55.85%)	
Yes	169 (5.90%)	1,095 (38.25%)	1,264 (44.15%)	
Skin flap reconstruction				<0.001
No	158 (5.52%)	2,349 (82.05%)	2,507 (87.57%)	
Yes	105 (3.66%)	251 (8.77%)	356 (12.43%)	
Number of operations				
Mean ± SD	1.37 ± 0.74	1.16 ± 0.47	1.18 ± 0.51	<0.01
Preoperative radiotherapy				<0.001
No	219 (7.65%)	2,428 (84.81%)	2,647 (92.46%)	
Yes	44 (1.53%)	172 (6.01%)	216 (7.54%)	
Age				0.235
Mean ± SD	60.39 ± 9.76	61.54 ± 10.02	61.44 ± 10.00	
Days of hospitalization				<0.001
Mean ± SD	21.57 ± 13.60	12.19 ± 7.06	13.05 ± 8.34	
Diabetes				<0.001
No	231 (8.07%)	2,408 (84.11%)	2,639 (92.18%)	
Yes	32 (1.11%)	192 (6.71%)	224 (7.82%)	
Hypertension				0.21
No	211 (7.37%)	2,171 (75.83%)	2,382 (83.20%)	
Yes	52 (1.82%)	429 (14.98%)	481 (16.80%)	
BMI				
Mean ± SD	21.83 ± 3.56	22.67 ± 3.75	22.59 ± 3.74	<0.01
Intraoperative bleeding				
Mean ± SD	80.67 ± 113.70	56.65 ± 81.12	58.86 ± 84.89	<0.001
Smoking				<0.001
No	199 (7.28%)	2,188 (80.03%)	2,387 (87.31%)	
Yes	46 (1.68%)	301 (11.01%)	347 (12.69%)	
Drinking				0.046
No	172 (6.29%)	1,735 (63.46%)	1,907 (69.75%)	
Yes	73 (2.67%)	754 (27.58%)	827 (30.25%)	
Preoperative antibiotics				<0.001
No	35 (1.25%)	855 (30.58%)	890 (31.83%)	
Yes	228 (8.15%)	1,678 (60.02%)	1,906 (68.17%)	
Duration of operation				
Mean ± SD	2.29 ± 0.74	1.92 ± 0.75	1.95 ± 0.76	<0.01
T stage				<0.001
T1	0 (0%)	670 (25.00%)	680 (25.00%)	
T2	50 (1.83%)	904 (33.24%)	954 (35.07%)	
T3	57 (2.10%)	398 (14.63%)	455 (16.73%)	
T4	86 (3.16%)	472 (17.35%)	558 (20.51%)	
Tis	0 (0%)	73 (2.68%)	73 (2.68%)	
N stage				<0.001
N0	83 (3.01%)	1,584 (57.37%)	1,667 (60.38%)	
N1	62 (2.25%)	467 (16.91%)	529 (19.16%)	
N2	88 (3.19%)	404 (14.63%)	492 (17.82%)	
N3	23 (0.83%)	50 (1.81%)	73 (2.64%)	
M stage				0.06
M0	189 (7.00%)	2,492 (92.30%)	2,681 (99.30%)	
M1	4 (0.15%)	15 (0.55%)	19 (0.70%)	

### Logistic regression analysis of factors associated with PCF

Univariable logistic regression was initially performed to evaluate associations between the candidate variables and the development of PCF after laryngectomy. The following variables were significantly associated with PCF in the univariable analyses (*P* < 0.05): neck dissection, skin flap reconstruction, number of operations, radiotherapy, chemotherapy, diabetes, intraoperative blood loss, T stage, N stage, smoking, preoperative antibiotic use, and duration of operation. These variables were subsequently entered into the multivariable logistic regression model.

In the multivariable logistic regression analysis, with postoperative PCF as the dependent variable, neck dissection, skin flap reconstruction, number of operations, diabetes, T3 stage, T4 stage, N2 stage, N3 stage, and duration of operation were independently associated with an increased risk of PCF after laryngectomy. The detailed results of the univariable and multivariable analyses are presented in Table 1.

### Prediction model development and performance evaluation

The dataset was stratified randomly divided into a training cohort comprising 80% of the participants and an internal validation cohort comprising the remaining 20%. Based on previous studies and clinical relevance, 17 candidate predictors were considered, including neck dissection, lymph node dissection, skin flap reconstruction, number of operations, preoperative radiotherapy, age, diabetes, hypertension, body mass index, intraoperative bleeding, smoking, drinking, preoperative antibiotic use, duration of operation, and T, N, and M stages. Five prediction models were developed in the training cohort: LR, RF, DT, XGB, and SVM. Their performance was subsequently evaluated in the internal validation cohort.

**Figure 2:**
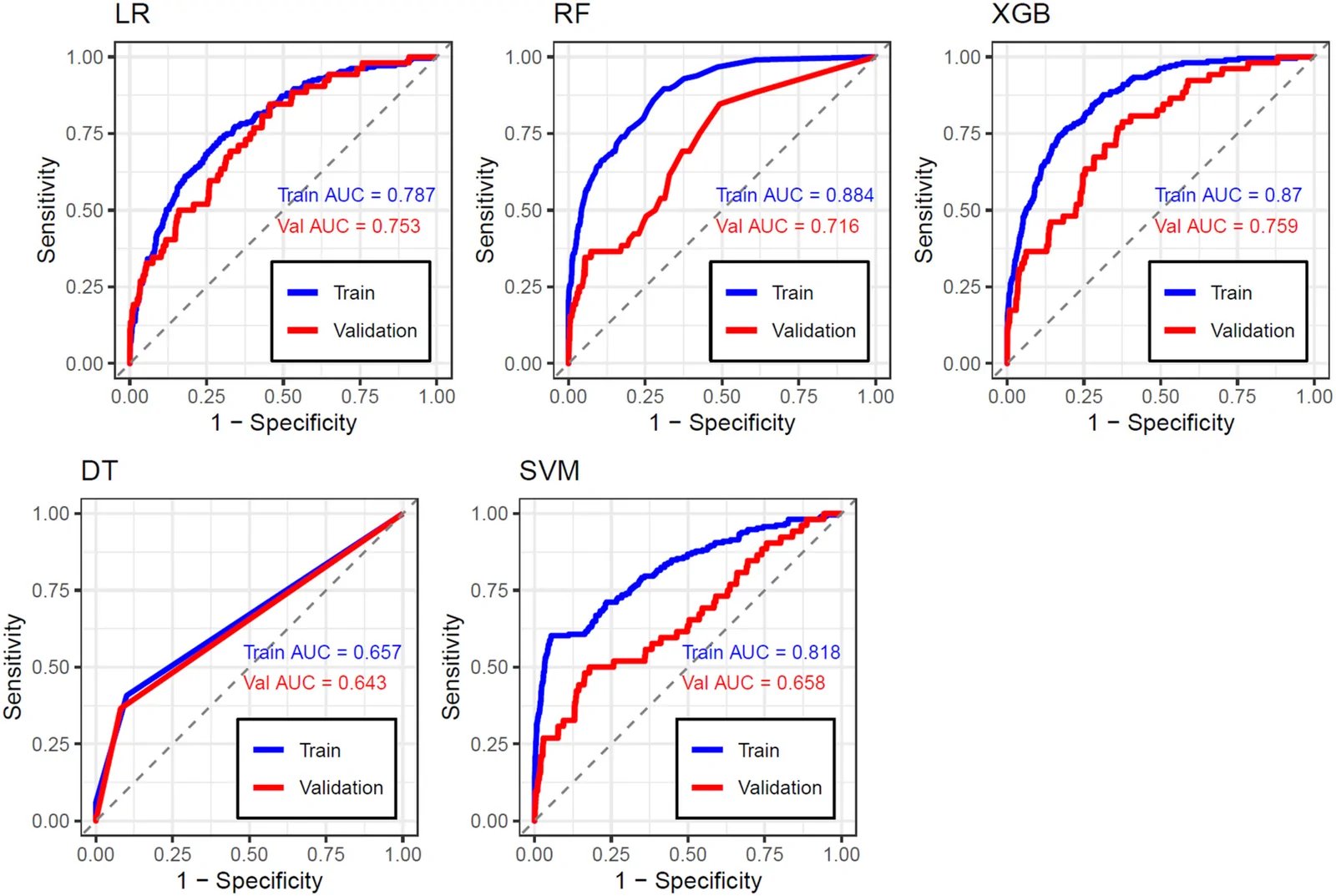
ROC curves for the five prediction models. The solid blue lines represent the ROC curves in the training cohort (80%) and the solid red lines represent the ROC curves in the validation cohort (20%). LR, logistic regression; RF, random forest; XGB, XGBoost; DT, decision tree; SVM, support vector machine. AUC values and 95% confidence intervals are provided.

As shown in Table 3 and Figure 2, the RF and XGB models achieved AUCs of 0.884 and 0.870 in the training cohort, respectively. However, their performance decreased in the validation cohort, with AUCs of 0.716 and 0.759, respectively. The SVM model also showed a decline, dropping from 0.818 in the training cohort to 0.658 in the validation cohort. In contrast, the LR model demonstrated relatively stable discrimination, with AUCs of 0.787 and 0.753 in the training and validation cohorts, respectively. The DT model exhibited the lowest discriminatory ability, with AUCs of 0.657 and 0.643 in the training and validation cohorts. Notably, XGB achieved the highest AUC (0.759) in the validation cohort, numerically surpassing LR (0.753).

**Table 3:** Comparison of model performance metrics between the training (80%) and validation (20%) cohorts.

Model	Dataset	AUC	Sensitivity	Specificity	Accuracy	Brier
LR	Train	0.787	0.735	0.705	0.708	0.072
LR	Validation	0.753	0.596	0.731	0.719	0.072
RF	Train	0.884	0.896	0.689	0.708	0.077
RF	Validation	0.716	0.615	0.671	0.666	0.084
XGB	Train	0.870	0.744	0.830	0.822	0.064
XGB	Validation	0.759	0.462	0.837	0.802	0.073
DT	Train	0.657	0.408	0.900	0.854	0.075
DT	Validation	0.643	0.365	0.919	0.869	0.076
SVM	Train	0.818	0.602	0.946	0.914	0.070
SVM	Validation	0.658	0.327	0.896	0.844	0.078

Metrics include the area under AUC-ROC, sensitivity, specificity, accuracy, and Brier score. AUC, sensitivity, specificity, and accuracy are presented as proportions ranging from 0 to 1; Brier scores are presented as absolute values. For interpretation as percentages, the corresponding values should be multiplied by 100.

In the validation cohort (Table 3), the DT model achieved the highest specificity among the evaluated models, at 91.9%, compared with 89.6% for the SVM model and 83.7% for the XGB model. However, the sensitivity of XGB was substantially lower than that of the LR and RF models (46.2% vs. 59.6% and 61.5%, respectively). Thus, while XGB generated fewer false-positive predictions, its lower sensitivity indicated a higher risk of false-negative predictions. In contrast, the LR model demonstrated a more balanced sensitivity–specificity profile (59.6% vs. 73.1%). The accuracy of the LR model was higher than that of the RF (71.9% vs. 66.6%).

**Figure 3:**
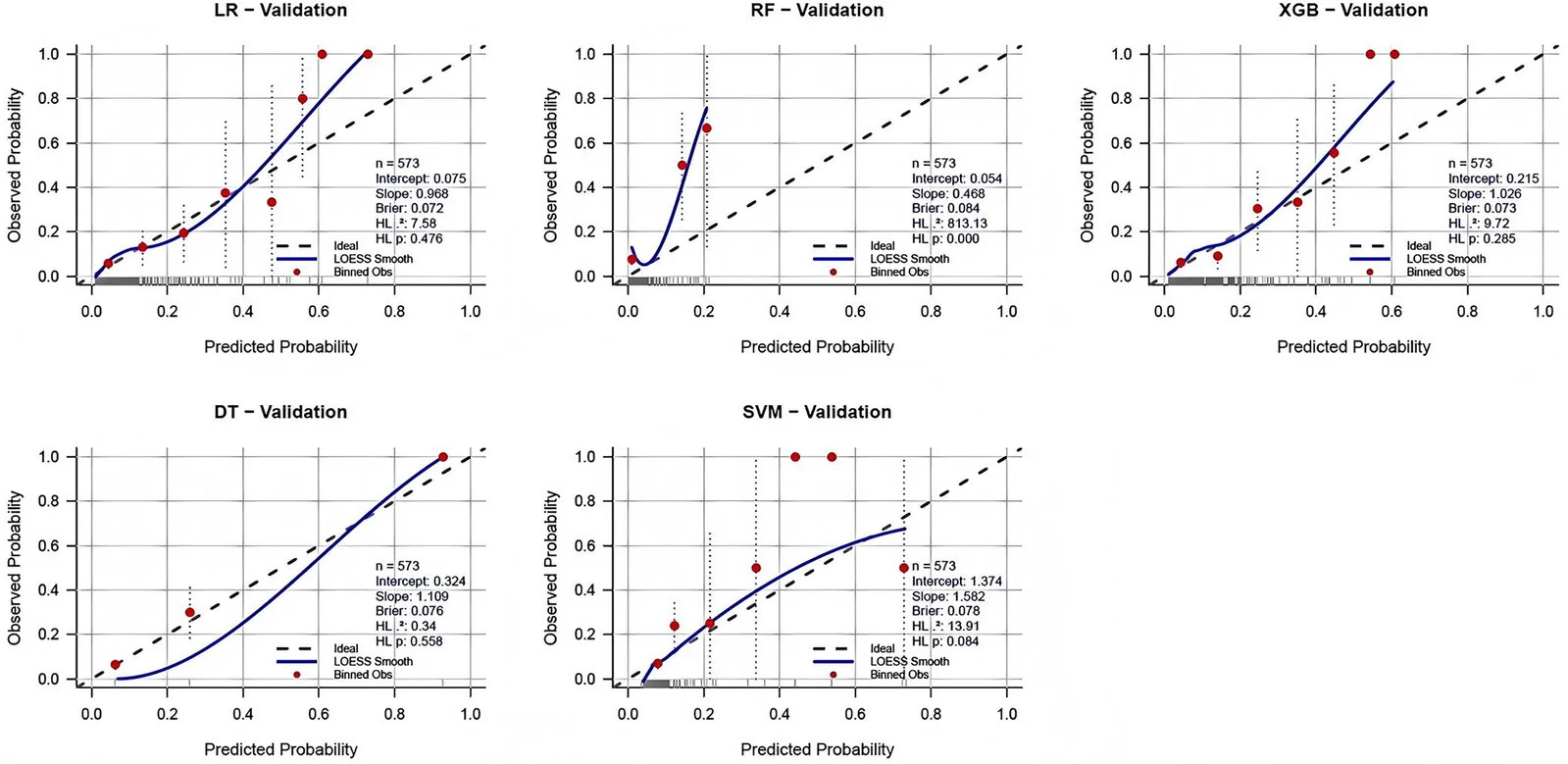
Calibration plots for the five models in the validation cohort. The *x*-axis represents the predicted probability of post-laryngectomy PCF and the *y*-axis represents the observed incidence. The dashed diagonal line indicates perfect calibration (i.e., predicted probability equals observed incidence). The solid blue line depicts the logistic calibration curve with loess-based non-parametric smoothing for each model. Among the five models, the Logistic Regression model exhibited the closest alignment with the ideal calibration line, suggesting superior calibration performance in the validation set.

Calibration was assessed using calibration curves and Brier scores (Figure 3 and Table 3). In the validation cohort, the LR calibration curve showed the closest agreement with the ideal calibration line. The Brier scores for LR, XGB, and RF were 0.072, 0.073, and 0.084, respectively. The RF calibration curve showed greater deviation from the ideal line, particularly at higher predicted probabilities. Both the XGB and SVM models also exhibited notable deviations between predicted and observed risks across the moderate-to-high predicted risk range.

**Figure 4:**
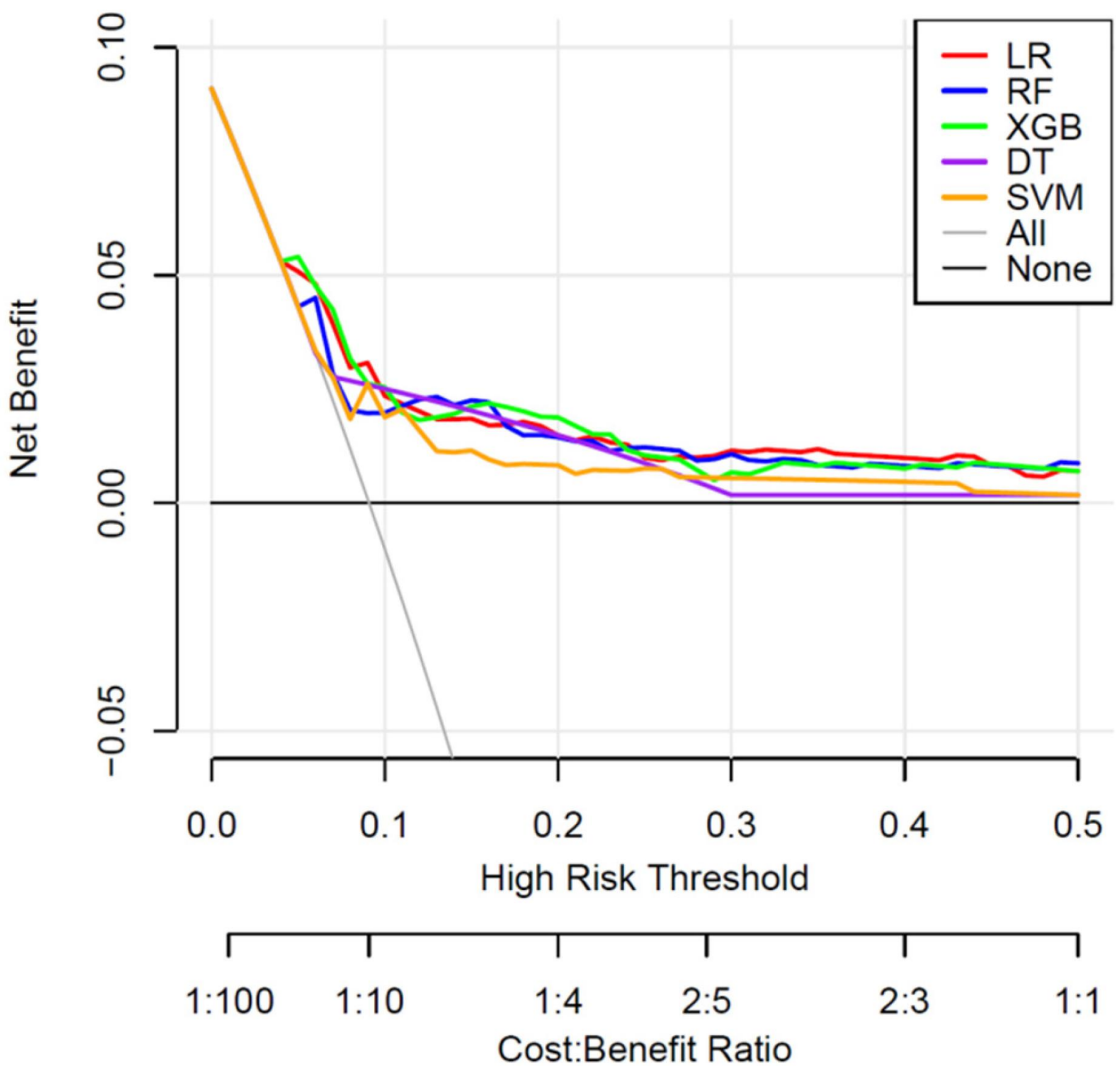
DCA for the five predictive models in the validation cohort. The *y*-axis represents net clinical benefit and the *x*-axis indicates the threshold probability. The “None” line assumes that no patients have PCF, whereas the “All” line assumes that all patients have PCF. The relative net benefit of the models varied across threshold probabilities; LR showed favorable performance over an intermediate range, while DT showed higher net benefit at some higher threshold probabilities.

Decision curve analysis was performed to assess the potential clinical utility of the models (Figure 4). The LR model showed a positive net clinical benefit across an intermediate range of threshold probabilities and generally performed favorably compared with RF and XGB in this range. The SVM model showed a higher net benefit at very low threshold probabilities, whereas the DT model showed a slightly lower net benefit at some higher threshold probabilities. These findings were considered together with model discrimination, calibration, classification performance, and validation performance when selecting the final model.

### Statistical comparisons of model discrimination

Pairwise comparisons of discrimination were performed using the DeLong test in the validation cohort. Table 4 presents the comparison of training and validation AUCs for each model, highlighting the generalization gaps (AUC_Difference). The RF, SVM, and XGB models showed the largest decreases in performance from training to validation (0.168, 0.160, and 0.111, respectively; both *P* < 0.001), raising concerns about overfitting. Table 5 details the pairwise comparisons among all models in the validation cohort. The AUCs of DT differed statistically from that of LR and XGB (*P* < 0.05), whereas no statistically significant difference was observed between LR and RF or between LR and XGB. Notably, although XGB yielded a numerically higher AUC than LR, this difference was not statistically significant (*P* = 0.722). These results suggest that the machine learning models did not provide a statistically superior discriminatory ability compared with the LR model.

**Table 4:** Comparison of ROC curves for the training and validation cohorts of each model.

Model	Train_AUC	Val_AUC	AUC_Difference	SE	Z	*P*-value	Significant
LR	0.787	0.753	0.034	0.0379	0.9032	0.367	No
RF	0.884	0.716	0.168	0.0387	4.3355	0.000	Yes
XGB	0.870	0.759	0.111	0.0358	3.0887	0.002	Yes
DT	0.657	0.643	0.014	0.0386	0.3491	0.727	No
SVM	0.818	0.658	0.160	0.0473	3.3854	0.001	Yes

We use the DeLong test to check the generalization ability of each model.

**Table 5:** Pairwise comparison of ROC curves using the DeLong test in the validation cohort.

Model 1	Model 2	AUC1	AUC2	AUC_Difference (Model 2 vs. Model 1)	SE	Z	*P*-value	*P*_adjusted (Bonferroni)	Significant
LR	RF	0.753	0.716	−0.037	0.023	1.568	0.117	1.000	No
LR	XGB	0.753	0.759	0.006	0.017	−0.356	0.722	1.000	No
LR	DT	0.753	0.643	−0.110	0.027	4.026	0.000	0.001	Yes
LR	SVM	0.753	0.658	−0.095	0.037	2.583	0.010	0.098	No
RF	XGB	0.716	0.759	0.043	0.023	−1.893	0.058	0.583	No
RF	DT	0.716	0.643	−0.073	0.029	2.491	0.013	0.127	No
RF	SVM	0.716	0.658	−0.058	0.037	1.573	0.116	1.000	No
XGB	DT	0.759	0.643	−0.116	0.028	4.128	0.000	0.000	Yes
XGB	SVM	0.759	0.658	−0.101	0.037	2.694	0.007	0.071	No
DT	SVM	0.643	0.658	0.015	0.039	−0.391	0.696	1.000	No

Model 1 was used as the reference baseline for comparisons. *P*-values were calculated to determine whether the AUC of each machine learning model differed significantly from that of Model 2.

### Final model selection and interpretation

The multivariable logistic regression results are presented in the preceding section. Considering discrimination, calibration, classification performance, clinical utility, and interpretability together, the LR model was selected as the final predictive model (Table 6). Although the XGBoost model showed a slightly higher area under the curve (AUC 0.759 vs. 0.753) in the validation cohort, this numerical advantage did not reach statistical significance (Z = −0.356, *P* = 0.722, adjusted *P* = 1.000, Table 6). Furthermore, XGB exhibited lower sensitivity (46.2%) and noticeable calibration deviations at moderate-to-high predicted risks. The RF model demonstrated a substantial performance drop between training and validation (AUC difference of 0.168, *P* < 0.001), raising concerns regarding its generalizability. Both DT and SVM performed significantly worse than LR in the validation set (adjusted *P* < 0.05).

**Table 6:** Comparison of ROC curves between the best model and other models.

Model	AUC	AUC_vs._Best	SE	Z	*P*-value	*P*_adjusted	Significant
LR	0.753	0.000	—	—	—	—	—
RF	0.716	−0.037	0.023	1.568	0.117	0.468	No
XGB	0.759	0.006	0.017	−0.356	0.722	1.000	No
DT	0.643	−0.110	0.027	4.026	0.000	0.000	Yes
SVM	0.658	−0.095	0.037	2.583	0.010	0.039	Yes

We use the LR model as a reference baseline for paired DeLong tests in the validation cohort.

The LR model demonstrated a more balanced sensitivity–specificity profile, favorable calibration, and positive net clinical benefit. Moreover, its transparent coefficient-based structure facilitates clinical interpretation and implementation. Therefore, the LR model was considered the preferred model for individualized prediction of postoperative pharyngocutaneous fistula.

## Discussion

In this study, logistic regression and machine learning models were developed to predict the occurrence of PCF after laryngectomy. Most models showed acceptable discrimination, with AUCs generally exceeding 0.700. Although some machine learning models achieved slightly higher AUCs than LR, these differences were not statistically significant. Therefore, the present findings do not provide sufficient evidence that machine learning algorithms offer superior predictive performance in this cohort. Similar results have been reported in previous studies comparing LR with machine learning models (11, 12).

Machine learning algorithms may offer several theoretical advantages over LR. For example, RF and XGB can capture non-linear relationships and potential interactions among predictors without requiring these effects to be specified in advance. Given the relatively limited number and diversity of conventional clinical predictors in the present study, simpler models such as LR may be more appropriate because of their stable performance, interpretability, and ease of clinical implementation. Larger, multicenter studies incorporating more diverse clinical, treatment-related, and perioperative variables are needed to determine whether machine learning methods provide clinically meaningful advantages for PCF prediction.

The comparison between training and validation performance underscored such concerns. RF and XGB achieved AUCs of 0.884 and 0.870 in the training cohort, but their AUCs decreased to 0.716 and 0.759, respectively, in the validation cohort. These substantial differences raised concerns about potential overfitting and limited generalizability. In contrast, LR showed relatively stable discrimination, with AUCs of 0.787 and 0.753 in the training and validation cohorts, respectively. Its Brier score was 0.072 and 0.072 in both cohorts, suggesting consistent predictive performance. Although XGB had a slightly higher validation AUC than LR, LR showed a smaller training–validation performance gap and a more favorable overall balance among stability, calibration, and interpretability. Therefore, LR was selected as the final model based on its overall performance and generalizability rather than on validation AUC alone.

Feature analysis identified skin flap reconstruction and advanced tumor stage—including T3, T4, N2, and N3 disease—as important contributors to postoperative PCF prediction. These findings are consistent with previous studies (13, 14). However, these variables should be interpreted as predictive features rather than definitive causal risk factors.

Skin flap reconstruction may reflect more extensive disease, larger surgical defects, or greater reconstructive complexity. Although it may be related to dead space formation, exudate accumulation, infection, and impaired wound healing, its association with PCF may also be influenced by confounding by indication. Likewise, advanced tumor stage may require wider resection and more complex reconstruction, thereby increasing the difficulty of wound healing. However, because the present study did not directly assess surgical defect size, the amount of viable mucosal tissue, or pharyngeal closure techniques, these proposed mechanisms could not be confirmed. Clinically, the presence of skin flap reconstruction and advanced tumor stage may help identify patients at higher predicted risk of PCF and may be considered when planning postoperative wound surveillance.

Overall, the present findings suggest that machine learning models and LR have comparable predictive performance in this cohort. Although machine learning models may provide a potential advantage in capturing complex relationships, their higher flexibility may also increase the risk of overfitting in relatively small datasets. In contrast, LR offers stable performance, transparent interpretation, and ease of clinical implementation. Further studies using larger, multicenter cohorts and independent external validation are needed to determine whether machine learning models can provide clinically meaningful improvements over LR in predicting PCF after laryngectomy.

### Limitations

This study has several limitations. First, this was a single-center retrospective study without external validation, which may limit the generalizability of the findings to other patient populations and clinical settings. Second, some potentially important clinical information, such as the use of preoperative radiotherapy or chemotherapy, was unavailable because of the retrospective study design. Although only variables available at or before the end of surgery were included in the prediction models, several important perioperative factors—including detailed surgical techniques, pharyngeal closure methods, reconstruction characteristics, surgeon experience, and postoperative management strategies—were not fully captured. In addition, confounding by indication may have affected the observed association between skin flap reconstruction and PCF, because skin flap reconstruction is more likely to be selected in patients with extensive disease, larger defects, previous radiotherapy, or more complex surgical conditions. These factors may influence PCF development and represent potential sources of residual confounding.

## Conclusion

In this single-center retrospective cohort, machine learning models did not significantly outperform multivariable logistic regression in discriminative performance. Given its interpretability, calibration, and ease of implementation, the logistic regression model may be preferable for initial clinical risk stratification. External validation and prospective impact studies are required before routine clinical implementation.

## Data Availability

The raw data supporting the conclusions of this article will be made available by the authors without undue reservation.
