# Supplementary material for: Comparison of multivariable logistic regression and machine learning models for predicting pharyngocutaneous fistula after surgery for laryngeal and hypopharyngeal carcinoma
Source: Front Surg. 2026 Sep 3;13:1867850. doi: 10.3389/fsurg.2026.1867850 (PMC13582574; doi:10.3389/fsurg.2026.1867850)
Supplement: Supplementary file 1 [file Table1.docx]

| **Supplementary Table S1**. Optimized Hyperparameters for Machine Learning Models | | | | |
| --- | --- | --- | --- | --- |
| Model | Algorithm | Hyperparameter | Value / Setting | Optimization Method |
| 1 | Logistic Regression | Variable selection threshold | VIF ≤ 10 for all 17 variables | Variance Inflation Factor |
| 1 | Logistic Regression | Estimation method | Maximum likelihood estimation | Preset |
| 2 | Random Forest | Number of trees (ntree) | 1000 | Preset |
| 2 | Random Forest | Number of variables per split (mtry) | Automatically selected via tuneRF | Tuning with 10-fold CV |
| 2 | Random Forest | Minimum node size (nodesize) | 20 | Preset (prevent overfitting) |
| 2 | Random Forest | Maximum nodes per tree (maxnodes) | 30 | Preset (prevent overfitting) |
| 2 | Random Forest | Sampling fraction (sampsize) | 70% of training set | Preset (bootstrap) |
| 2 | Random Forest | Replacement | Yes | Preset |
| 3 | XGBoost | Learning rate (eta) | 0.03 | Preset |
| 3 | XGBoost | Maximum tree depth (max_depth) | 4 | Preset |
| 3 | XGBoost | Minimum child weight (min_child_weight) | 5 | Preset |
| 3 | XGBoost | Subsample ratio | 0.8 | Preset |
| 3 | XGBoost | Column subsample per tree (colsample_bytree) | 0.7 | Preset |
| 3 | XGBoost | Column subsample per level (colsample_bylevel) | 0.7 | Preset |
| 3 | XGBoost | L1 regularization (alpha) | 1 | Preset |
| 3 | XGBoost | L2 regularization (lambda) | 2 | Preset |
| 3 | XGBoost | Minimum loss reduction (gamma) | 0.1 | Preset |
| 3 | XGBoost | Number of boosting rounds (nrounds) | Optimized by early stopping (50 rounds) | 10-fold CV with early stopping |
| 3 | XGBoost | Evaluation metric | AUC | Preset |
| 4 | Decision Tree | Minimum split size (minsplit) | 20 | Preset |
| 4 | Decision Tree | Minimum leaf node size (minbucket) | 7 | Preset |
| 4 | Decision Tree | Maximum depth (maxdepth) | 10 | Preset |
| 4 | Decision Tree | Complexity parameter (cp) | Optimized via cost-complexity pruning | 10-fold CV (minimum xerror) |
| 5 | SVM (RBF kernel) | Kernel type | Radial basis function | Preset |
| 5 | SVM (RBF kernel) | Cost parameter (C) | Optimized via grid search (10⁻¹ to 10²) | 10-fold CV |
| 5 | SVM (RBF kernel) | Kernel parameter (gamma) | Optimized via grid search (10⁻³ to 10¹) | 10-fold CV |
| 5 | SVM (RBF kernel) | Convergence tolerance | 0.001 | Preset |
| 5 | SVM (RBF kernel) | Shrinking heuristic | Enabled | Preset |

CV, cross-validation; VIF, variance inflation factor; AUC, area under the receiver operating characteristic curve; RBF, radial basis function.
